# Acute Liver Failure following a Single Dose of Atezolizumab, as Assessed for Causality Using the Updated RUCAM

**DOI:** 10.1155/2022/5090200

**Published:** 2022-03-23

**Authors:** Roie Tzadok, Sharon Levy, Jessie Aouizerate, Oren Shibolet

**Affiliations:** ^1^Department of Internal Medicine C, Tel Aviv Sourasky Medical Center, Tel Aviv, Israel; ^2^Department of Gastroenterology and Hepatology, Tel Aviv Sourasky Medical Center and the Sackler Faculty of Medicine, Tel Aviv University, Tel Aviv, Israel; ^3^Institute of Pathology, Tel Aviv Sourasky Medical Center, Tel Aviv, Israel

## Abstract

Immune checkpoint inhibitors have become major therapeutic agents in oncology over the last few years. However, they are associated with a variety of potentially severe autoimmune phenomena. We present a patient with advanced adenocarcinoma of the lung, who presented with acute liver injury two weeks following his first treatment with atezolizumab, rapidly deteriorating to fulminant liver failure. A thorough evaluation of infectious, vascular, metabolic, and autoimmune etiologies did not yield any results. Liver pathology was nonspecific. Using RUCAM as a causality assessment method indicated probable connection between atezolizumab and liver damage. To our knowledge, this is the first documented report of a patient developing acute liver failure shortly after immune checkpoint inhibitor initiation.

## 1. Introduction

Immune checkpoint inhibitors (ICI) are antineoplastic agents that activate the immune system against tumor cells. Programmed death 1 (PD-1), its ligand-1 (PD-L1), and cytotoxic T-lymphocyte antigen 4 (CTLA-4) are the most common targets of these agents, which operate by relieving tumor-induced T-cell inactivation [1]. Atezolizumab is an Fc-modified IgG1 humanized monoclonal antibody directed against PD-L1 [2]. In the past several years, immunotherapy with ICI has become a leading treatment strategy in oncology, improving response and overall survival rates in a variety of neoplastic diseases [3].

Although ICI are usually well tolerated, their incurred immune activation may lead to autoimmune-like phenomena affecting a variety of organs, including the liver, colon, lungs, pituitary gland, thyroid, and skin. The extent of tissue injury varies but may be severe and even life threatening [4, 5]. All ICI induce autoimmunity, but ipilimumab, a CTLA-4 inhibitor, as well as combinations of ICI are considered more hepatotoxic than other ICI monotherapy regimens [6]. Although the mechanism for this immune-mediated toxicity is still unclear, antigen similarity between tumor cells and native host tissues may play a role [7]. Risk factors contributing to increased risk of ICI-induced liver injury may be the use of higher doses, a preexisting tendency to autoimmunity unmasked by ICI use or a preexisting liver disease [3].

## 2. Case Presentation

A 46-year-old male was diagnosed with adenocarcinoma of the lung and was treated with neo-adjuvant chemoradiation and lobectomy but was diagnosed with disease recurrence. His disease involved the right lung, cervical and thoracic vertebrae, pleura, mediastinal lymph nodes, and adjacent ribs. He was treated with a single-agent immunotherapy protocol with atezolizumab (without other coadministered drugs).

About two weeks after receiving the first treatment, he presented to our hospital complaining of weakness, vomiting, and epigastric pain in the previous week. He reported sporadic consumption of paracetamol a few days prior to his presentation, well below the considered hepatotoxic range. His physical examination was unremarkable. Laboratory tests demonstrated stable normocytic anemia with a hemoglobin of 8.1 g/dL, 9,400 leukocytes with an absolute lymphocyte count of 300 (10^3^/*μ*L), and 311,000 platelets. The sodium level was 125 mmol/L (normal 135–145 mmol/L), creatinine 1.37 (normal 0.7–1.3 mg/dL), and bilirubin 0.3 (normal 0.1–1.2 mg/dL). A new-onset mixed pattern of liver enzymes elevation was noted, with alanine aminotransferase (ALT) of 485 (normal 8–40 U/L), aspartate aminotransferase (AST) of 253 (normal 7–40 U/L), gamma glutamyltransferase (GGT) of 560 (normal 6–42 U/L), and alkaline phosphatase (ALKP) of 261 (normal 46–116 U/L). The international normalized ratio (INR) was 1.32 (normal 0.97–1.19). Urine tests indicated hypovolemic hyponatremia, most probably due to excessive vomiting. Abdominal and brain imaging ruled out bowel obstruction or intracranial metastases as causes of vomiting.

The patient was hospitalized in stable condition without hypotension and was treated with fluids. Over the next five days, he continued to deteriorate rapidly and progressed from acute liver injury to fulminant liver failure, with new-onset encephalopathy, further prolongation of INR to 2.91, and acute kidney injury with creatinine elevation up to 2.8 mg/dL, with accompanying profound lactic acidosis. His liver test abnormalities progressed with a predominant hepatocellular pattern (*R* = 25) [8], with ALT up to 2124 U/L and AST 1726 U/L. Cholestatic enzymes and bilirubin remained stable, and glucose levels were within the normal range.

**Figure 1:**
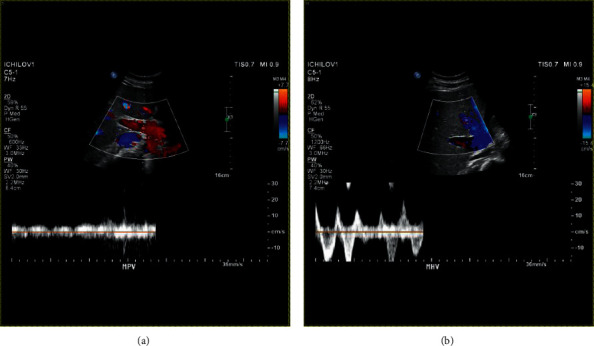
Liver Doppler sonography showing normal venous flow in the main portal vein (a) and three hepatic veins (b).

The patient was not admitted to an intensive care unit (ICU) but was managed in an internal medicine department. He did not need mechanical ventilation at any stage. During his hospitalization, he underwent a full evaluation to determine the cause of his deterioration. Blood and urine cultures were negative. Liver ultrasonography and Doppler did not demonstrate any focal hepatic or vascular findings (portal vein thrombosis or Budd-Chiari syndrome) (Figure 1). A thorough evaluation for infectious, autoimmune, and metabolic etiologies of acute liver failure yielded no results: viral hepatitis (hepatitis B surface antigen and antibody, hepatitis B core antibody, hepatitis C antibody, and hepatitis A IgG antibody) and HIV serologies were negative. Ethanol levels were not elevated. CMV, EBV, VZV, and HSV serologies indicated past infections. Toxoplasma and celiac serologies were negative. Immunoglobulin levels were normal. Autoantibodies including ANA, ASMA, AMA, and anti-LKM were normal.

**Figure 2:**
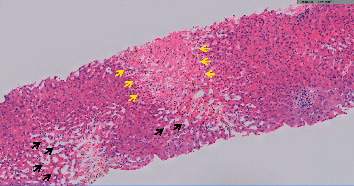
Liver biopsy showing centrilobular coagulative necrosis (yellow arrows) with marked sinusoidal dilatation (black arrows). No significant inflammatory infiltrate is seen.

He developed large volume ascites and underwent paracentesis, demonstrating high serum-ascites albumin gradient (19 g/L) and ascites protein content of 24 g/L, suggestive of portal hypertension secondary to a hepatic cause. The patient also underwent a percutaneous liver biopsy (Figure 2), which showed zone 3 (centrilobular) necrosis and mild steatosis with bridging necrosis and mild neutrophilic infiltrate. Sinusoidal dilatation was also noted. No fibrosis was seen.

Hepatic sinusoidal dilatation is usually caused by hepatic venous outflow obstruction, whether due to pericardial disease, congestive heart failure, or direct hepatic vascular involvement (as in Budd-Chiari syndrome or sinusoidal obstruction syndrome). It may also happen in extrahepatic inflammatory diseases, without venous outflow obstruction. In these cases, the mechanism is not as clear but may be attributed to hemodynamic changes and overexpression of interleukin-6 (IL-6) [9]. The biopsy was interpreted as nonspecific, but a differential diagnosis of drug-induced liver injury (DILI) was suggested. Because there were no documented hypotensive episodes and blood cultures were sterile, we initiated empirical pulse steroid therapy (1000 mg methylprednisolone daily) and N-acetylcysteine after the biopsy was given. Due to the patient's metastatic disease, liver transplantation was not discussed.

Due to advancing acute liver injury with oliguria and acidosis, which was suspected to be checkpoint inhibitor-induced acute interstitial nephritis, the patient was scheduled to undergo hemodialysis. However, he developed severe shock and died after five days.

## 3. Discussion

This is a unique case of acute liver failure in a patient with metastatic adenocarcinoma of the lung, which developed shortly after one course of atezolizumab. Rigorous attempts to find other etiologies yielded no results. The pathologic report suggested DILI. The differential diagnosis of ischemic hepatitis seems less relevant as no vascular pathology was noted on Doppler sonography, and no hypotensive events were noted. Right heart failure leading to hepatic congestion and dilated sinusoids, possibly as a result of checkpoint-induced myocarditis, a previously described toxic side effect of ICI [10, 11], was also considered. The patient did not undergo echocardiography, but his physical examination and chest X-ray showed no signs of congestion, and his electrocardiogram demonstrated sinus rhythm without arrhythmias or ischemic changes. Therefore, a cardiac etiology for his condition seems less likely.

The Roussel Uclaf Causality Assessment Method (RUCAM) is a scale based on seven category parameters to assess causality for a liver injury being due to a specific medication. As the patient described in this case presented with a pattern of hepatocellular injury (R value > 5), the relevant RUCAM score was calculated to be +7, indicating atezolizumab to be a probable cause of liver failure. Alternatively, the RUCAM score for causality of acetaminophen-induced injury is +4, indicating only a possible connection, which is not supported by the clinical story [8].

Hepatic side effects have been reported in 3–10% of patients with ICI [12, 13] and most commonly manifest as asymptomatic elevations of liver enzymes, mostly transaminases, and mildly elevated bilirubin [14]. This pattern of liver enzyme abnormality was also present in this case.

Grading the severity of hepatic damage is based on the Common Terminology Criteria for Adverse Events (CTCAE) established by the Cancer Therapy Evaluation Program of the National Cancer Institute and is based on levels of multiples of the upper normal limit of ALT, AST, ALKP, GGT, and bilirubin [15]. Therefore, as in this case, a patient may be classified as grade 4 liver injury without concomitant bilirubin elevation.

The median time of onset of immune-mediated hepatitis is 6–14 weeks from treatment initiation, after a median of 3 doses of immunotherapy. However, it may also occur at a much later phase, or even after therapy cessation [3, 6]. To our knowledge, this is the first documented report of a patient developing acute liver failure shortly after ICI initiation. A single case of nivolumab-induced hepatitis in a patient with renal cell carcinoma which developed within two weeks of therapy initiation was previously reported but was not as clinically severe [16].

In a single-center retrospective analysis of 17 patients with immune ICI-induced hepatotoxicity, Huffman et al. showed that about half of the patients also had concurrent immune-related adverse events in other systems [17]. Our patient also suffered from acute renal failure, which was suspected to be therapy-induced acute interstitial nephritis, a well-documented renal adverse effect of ICI [18].

Pathological reports of anti-PD-1-related hepatic injury described a heterogeneous histological picture, with lobular hepatitis, scattered foci of necrosis, and sometimes lobular fibrosis [14, 19]. These histologic findings are not specific and may also be found in other conditions, such as autoimmune hepatitis (AIH), viral hepatitis, and DILI secondary to various agents. The differentiation from AIH may be particularly difficult: elevated IgG levels and typical autoantibodies, as well as an inflammatory infiltrate of B cells and CD4+ T cells on biopsy, may be suggestive of AIH, whereas temporal association with administration of the ICI and a sinusoidal distribution of histiocytic and CD8+ T-cell infiltration may imply ICI-induced hepatitis. A mixed inflammatory infiltrate containing neutrophils, as described here, is also a common finding [2, 14, 20].

Apart from stopping the offending agent, there is currently no approved therapy for DILI. Corticosteroids are often given, but their benefit is unknown. In a recently published systematic review, the majority of studies suggested a beneficial effect to steroids in moderate to severe DILI, while some showed no effect. Steroids have not shown improvement in survival in drug-induced fulminant hepatic failure. In ICI-induced DILI, they have shown a beneficial effect. Thus, although the data are inconclusive, steroids are routinely given in DILI and in some cases may be beneficial [21].

In conclusion, we report a rare case of acute liver failure after only one infusion of atezolizumab. An extensive work-up for other etiologies was negative.

Although exceptional, the increasing use of ICI for a widening spectrum of neoplastic diseases warrants physicians' awareness of the wide variety of hepatic manifestations, ranging from mild transient enzyme elevation to autoimmune hepatitis and, as shown in this case, to fulminant hepatic failure leading to rapid deterioration and fatal consequences.

## Data Availability

All data are recorded at the Tel Aviv Sourasky Medical Center and are subject to patient confidentiality regulations.

## References

[1] Iranzo I., Huguet J. M., Suárez P., Ferrer-Barceló L., Iranzo V., Sempere J. (2018). Endoscopic evaluation of immunotherapy-induced gastrointestinal toxicity. *World Journal of Gastrointestinal Endoscopy*.

[2] Vitale G., Lamberti G., Comito F. (2020). Anti-programmed cell death-1 and anti-programmed cell death ligand-1 immune-related liver diseases: from clinical pivotal studies to real-life experience. *Expert Opinion on Biological Therapy*.

[3] EASL Clinical Practice Guidelines (2019). Drug-induced liver injury. *Journal of Hepatology*.

[4] Wang D. Y., Salem J.-E., Cohen J. V. (2018). Fatal toxic effects associated with immune checkpoint inhibitors. *JAMA Oncology*.

[5] Wang Y., Zhou S., Yang F. (2019). Treatment-related adverse events of PD-1 and PD-L1 inhibitors in clinical trials. *JAMA Oncology*.

[6] Wang W., Lie P., Guo M., He J. (2017). Risk of hepatotoxicity in cancer patients treated with immune checkpoint inhibitors: a systematic review and meta-analysis of published data. *International Journal of Cancer*.

[7] Berner F., Bomze D., Diem S. (2019). Association of checkpoint inhibitor-induced toxic effects with shared cancer and tissue antigens in non-small cell lung cancer. *JAMA Oncology*.

[8] Danan G, Teschke R (2015). RUCAM in drug and herb induced liver injury: the update. *International Journal of Molecular Sciences*.

[9] Brancatelli G., Furlan A., Calandra A., Dioguardi Burgio M. (2018). Hepatic sinusoidal dilatation. *Abdominal Radiology*.

[10] Mahmood S. S., Fradley M. G., Cohen J. V. (2018). Myocarditis in patients treated with immune checkpoint inhibitors. *Journal of the American College of Cardiology*.

[11] Varricchi G., Galdiero M. R., Marone G. (2017). Cardiotoxicity of immune checkpoint inhibitors. *ESMO open*.

[12] Cramer P., Bresalier R. S. (2017). Gastrointestinal and hepatic complications of immune checkpoint inhibitors. *Current Gastroenterology Reports*.

[13] Weber J. S., Kähler K. C., Hauschild A. (2012). Management of immune-related adverse events and kinetics of response with ipilimumab. *Journal of Clinical Oncology*.

[14] Karamchandani D. M., Chetty R. (2018). Immune checkpoint inhibitor-induced gastrointestinal and hepatic injury: pathologists’ perspective. *Journal of Clinical Pathology*.

[15] Services Usdohah Common terminology criteria for adverse events (CTCAE). https://ctep.cancer.gov/protocoldevelopment/electronic_applications/docs/ctcae_v5_quick_reference_5x7.pdf.

[16] Mathew Thomas V., Bindal P., Ann Alexander S., McDonald K. (2020). Nivolumab-induced hepatitis: a rare side effect of an immune check point inhibitor. *Journal of Oncology Pharmacy Practice*.

[17] Huffman B. M., Kottschade L. A., Kamath P. S., Markovic S. N. (2018). Hepatotoxicity after immune checkpoint inhibitor therapy in melanoma. *American Journal of Clinical Oncology*.

[18] Wanchoo R., Karam S., Uppal N. N. (2017). Adverse renal effects of immune checkpoint inhibitors: a narrative review. *American Journal of Nephrology*.

[19] De Martin E., Michot J.-M., Papouin B. (2018). Characterization of liver injury induced by cancer immunotherapy using immune checkpoint inhibitors. *Journal of Hepatology*.

[20] Zen Y., Yeh M. M. (2019). Checkpoint inhibitor-induced liver injury: a novel form of liver disease emerging in the era of cancer immunotherapy. *Seminars in Diagnostic Pathology*.

[21] Björnsson E. S., Vucic V., Stirnimann G., Robles-Díaz M. (2022). Role of corticosteroids in drug-induced liver injury. A systematic review. *Frontiers in Pharmacology*.

